# Treatment outcomes of extrapulmonary tuberculosis in Bahawalpur, Pakistan; a record review

**DOI:** 10.1186/s40545-020-00227-1

**Published:** 2020-07-24

**Authors:** Muhammad Atif, Razia Fatima, Nafees Ahmad, Zaheer-Ud-Din Babar

**Affiliations:** 1 Department of Pharmacy, The Islamia University of Bahawalpur, Bahawalpur, Punjab Pakistan; 2 Research Unit, National TB Control Program, Islamabad, Pakistan; 3 Department of Pharmacy, University of Baluchistan, Quetta, Pakistan; 4 Department of Pharmacy, University of Huddersfield, Huddersfield, UK

**Keywords:** Extrapulmonary tuberculosis, Treatment outcomes, Unsuccessful treatment outcome, Unfavorable treatment outcome, High TB burden countries, Pakistan

## Abstract

**Background:**

There is limited published data form Pakistan on treatment success rate among extrapulmonary tuberculosis (EPTB) patients. The aim of this study was to assess clinical form, treatment outcomes, and identify the factors associated with unfavorable treatment outcome among EPTB patients.

**Methods:**

A retrospective study was conducted at the Chest Disease Unit of the Bahawal Victoria Hospital, Pakistan. Medical records of EPTB patients, registered at the study site from January 1, 2015 to September 30, 2017, were reviewed to obtain the data. Final treatment outcomes among EPTB patients were evaluated in accordance with the standard Word Health Organization (WHO) criteria. Multivariate binary logistic regression analysis was used to identify the factors associated with unfavorable treatment outcome.

**Results:**

A total of 651 EPTB patients were included in the study. Highest proportion of patients had pleural TB (*n* = 217, 33.3%). Out of the total 651 patients, 463 (71.1%) successfully completed the treatment. Among 177 (27.2%) patients with unfavorable treatment outcome, 10 (1.5%) died, while 165 (25.4%) lost to follow-up the treatment. Lymph node TB (AOR 0.65, 95% CI 0.422, 0.989) and meningeal TB (AOR 2.1, 95% CI 1.065, 4.144) were significantly associated with unfavorable treatment outcome.

**Conclusion:**

The treatment success (favorable outcome) rate among EPTB patients was less than the target (i.e., ≥ 90%) set by the WHO. Highest proportion of patients lost to follow-up during the treatment.

## Introduction

Tuberculosis (TB) is an infectious disease caused by *Mycobacterium tuberculosis.* It is primarily a disease of the lungs (i.e., pulmonary TB), but can also affect other organs of the body (i.e., extrapulmonary TB) [1]. According to the Global Tuberculosis Report (2018), 10 million people developed TB disease and 1.3 million died (Human Immunodeficiency Virus negative) from this illness in 2017 [2]. Available evidences showed that an increase in the number of newly diagnosed EPTB cases were observed worldwide [1, 3, 4]. In the Eastern Mediterranean Region, the percentage of EPTB case notifications increased from 22.9% in 2014 to 24% in 2017 [2, 4]. Pakistan faced an incidence rate of 267 cases of TB per 100,000 population (all forms TB cases) [2] while the percentage of EPTB cases notified in the country raised from 15.4% in 2012 to 20% in 2017 [2, 4, 5]. Increase in the burden of EPTB in recent years could have a significant impact on the national health resources.

Reporting treatment outcomes among TB patients is one of the major indicators to evaluate the performance of national tuberculosis control program (NTP) [6]. The International Union against Tuberculosis and Lung Disease and the World Health Organization (WHO) jointly published recommendations to standardize the reporting of treatment outcomes among TB patients. Though, these recommendations are aimed at pulmonary tuberculosis (PTB) but are also used for EPTB [7]. In recent years, countries such as Nigeria (52.3%), Ethiopia (79.2%) and India (78.1%) documented suboptimal treatment success rates among EPTB patients [8–10]. In contrast, similar studies from Bhutan (90%) and India (90.5%) reported better treatment success rates [11, 12]. Earlier studies reported age, gender, type of patient, Human Immunodeficiency Virus (HIV) status and diabetes as most common factors associated with treatment success rate among EPTB patients [7, 11].

In Pakistan, the treatment success rate for TB (all forms) was 94% (2017 cohort) [2]. A few studies from Pakistan reported 60–88% treatment success rate among EPTB patients [13, 14]. Nevertheless, to-date, limited data is available on clinical form and treatment outcomes among EPTB patients. In particular, it remains unclear what are the most common risk factors associated with unfavorable treatment outcome among EPTB patients. Therefore, the aim of this study was to assess clinical form, treatment outcomes and identify the factors associated with unfavorable treatment outcome among EPTB patients at the Bahawal Victoria Hospital (BVH), Punjab, Pakistan.

## Methods

### Study design

This was a retrospective record review [15–17] of EPTB patients registered at the study site from 2015 to 2017.

### Study setting

The study was conducted in the Bahawalpur district of the Punjab province of Pakistan. Bahawalpur is the 12th largest city of Pakistan with an approximate population of 3,333,467 people. There are two public sector tertiary healthcare facilities in Bahawalpur i.e., the BVH and the Civil Hospital. This study was undertaken at the Chest Disease Unit (CDU) of the BVH. The BVH, a 1600 bedded health facility, is a referral tertiary care hospital located in the southern region of the Punjab province of Pakistan. The CDU of the BVH has 8–10 physicians, 5–6 chest specialists and two pharmacists who provide routine care to the patients with chest-related diseases. In addition, TB outdoor clinic has dedicated paramedic staff committed to provide quality care to TB patients. TB outdoor clinic is visited by 35–40 TB patients daily. TB unit in the chest clinic works under the NTP [18].

Presumptive cases of EPTB are often diagnosed on microbiological and/or clinical grounds [19]. After being diagnosed with EPTB, patients are provided with standard anti-TB regimen comprising of isoniazid (H), rifampicin (R), pyrazinamide (Z) and ethambutol (E) for 2 months followed by isoniazid (H) and rifampin (R) for 4 months (2HRZE/4HR). Re-treatment cases are treated with 3HRZE/6HRE. Duration of treatment in meningeal TB patients may be as long as 20 months. The patients who default form the treatment for more than 4 weeks are contacted via telephone, and traced by a TB-coordinator.

### Patients and data collection

The study included all new and retreatment EPTB patients aged ≥15 years diagnosed and registered at the study site from January 1, 2015 to September 30, 2017. During May and June 2018, patient files were reviewed to collect socio-demographic, clinical and treatment-related data on age, sex, distance from the treatment center, body weight, type of patient, site of disease, diagnostic tests and final treatment outcomes [20, 21].

### Reporting of treatment outcomes (main outcome variable)

The outcome of TB treatment was reported in accordance with the standard criteria [22]. Treatment failure was defined as, “a patient whose treatment fails based on clinical judgment of the physician, for example, size of lymph node, radiographic findings, colonoscopy etc.” [23–25]. Treatment completed was further classified as favorable treatment, whereas treatment failure, died and loss to follow-up were classified as unfavorable treatment outcome [26]. Table 1 outlines the standard definitions of treatment outcomes.

**Table 1:** Definition of treatment outcomes among extrapulmonary tuberculosis patients

Outcome	Definition
Treatment completed	A TB patient who completed treatment without evidence of failure but with no record to show that sputum smear or culture results in the last month of treatment and on at least one previous occasion were negative, either because tests were not done or because results are unavailable.
Treatment failure	Patients who were initially diagnosed as EPTB (with or without bacteriological confirmation), and those started on treatment based on clinical and radiological findings, who have not shown clinical improvement or became smear (or culture) positive or their condition deteriorated during the course of treatment. This definition excludes those patients who are diagnosed with RR-TB or MDR-TB during treatment.
Died	A TB patient who dies for any reason before starting or during the course of treatment.
Loss to follow-up	A TB patient who did not start treatment or whose treatment was interrupted for two consecutive months or more.
Not evaluated	A TB patient for whom no treatment outcome is assigned. This includes cases “transferred out” to another treatment unit as well as cases for whom the treatment outcome is unknown to the reporting unit.
Favorable treatment outcome	Patients categorized as treatment completed.

Adapted from: Definitions and reporting framework for tuberculosis – 2013 revision World Health Organization; National Tuberculosis Management Guidelines, Republic of South Africa (2014).

### Statistical analysis

Data were double entered in EpiData (version 3.1 for entry, EpiData Association, Odense, Denmark) and analyzed using the Statistical Package for Social Sciences (IBM, SPSS Statistics for Windows, version 21.0. Armonk, NY: IBM Corp.). Categorical variables were presented as counts and proportions (%). Continuous variables were described as mean and standard deviations (SD). Simple logistic regression analysis was used to evaluate the relationship between the dependent variable (i.e., unfavorable treatment outcome) and the selected independent variables. Statistically significant variables in univariate analysis were analyzed using multivariate binary logistic regression analysis to find the independent factors associated with unfavorable treatment outcome. The adjusted odd ratios (AOR) and 95% CI was documented for each variable. The significance of the statistical tests was taken at a *p*-value of < 0.05.

*Note:* In this study, “successful treatment outcome” and “favorable treatment outcome” are used interchangeably, and are similar in meaning.

## Results

During the study period, a total of 723 EPTB patients were registered at the study site. Out of these, medical records of 72 (10%) patients were not traceable due to assorted reasons. Consequently, 651 patients were included in the study. Among these, 648 (99.5%) were new patients and only 3 (0.5%) were retreatment case. The mean age of the patients was 33.7 (SD = 14.9) years. Close to half of the patients (*n* = 317; 48.7%) were female (Table 2).

**Table 2:** Socio-demographic characteristics of extrapulmonary tuberculosis patients registered at Bahawal Victoria Hospital, Bahawalpur from 2015 to 2017 (*N* = 651)

Characteristics	Patients n (%)
**Sex**	
Male	334 (51.3)
Female	317 (48.7)
**Age group (years)** mean age = 33.7 (SD = 14.9)	
15–24	232 (35.6)
25–34	171 (26.3)
35–44	92 (14.2)
45–54	75 (11.5)
55–64	45 (6.9)
≥ 65	36 (5.5)
**Distance from the treatment center (Km)**	
< 5	87 (13.4)
6–25	253 (38.9)
26–50	204 (31.3)
> 50	107 (16.4)

With regard to site of infection, highest proportion of patients had pleural TB (n = 217, 33.3%) followed by lymph node TB (*n* = 170, 26.1%). More than 65% (*n* = 437; 67.1%) of the patients had their histopathological tests done, while basis of diagnosis was not recorded in 15 (2.3%) patients (Table 3.)

**Table 3:** Site of infection and diagnostic attributes of extrapulmonary tuberculosis patients registered at Bahawal Victoria Hospital, Bahawalpur from 2015 to 2017 (*N* = 651)

Clinical Characteristics	Patients n (%)
**Site of infection**	
Pleural	217 (33.4)
Lymphatic	170 (26.1)
Abdominal/ascites	80 (12.3)
Bone/joint/spinal	23 (3.5)
Meningeal	38 (5.8)
Skin	6 (0.9)
Other ^a^/not recorded	117 (18)
**Diagnostic test**	
Histopathology	437 (67.1)
CT scan/MRI	67 (10.3)
X-ray	409 (62.8)
Ultrasonography	76 (11.7)
Other ^b^	10 (1.5)
Not recorded/not done	15 (2.3)

^a^ Testicular, Genitourinary, other organ involvement, ^b^ ESR, CBC, LFT, RFT, CT = Computed Tomography, MRI = Magnetic Resonance Imaging

Table 4 describes treatment outcomes among EPTB patients. Out of the total 651 patients, 463 (71.1%) completed the treatment (i.e., favorable treatment outcome). Among 177 (27.2%) patients with unfavorable treatment outcome, 10 (1.5%) died and 165 (25.4%) lost to follow-up during the treatment. A small proportion of the patients (*n* = 11, 1.7%) were not evaluated for the treatment outcome because their final treatment outcomes were not recorded in the medical records.

**Table 4:** Treatment outcomes among extrapulmonary tuberculosis patients registered at Bahawal Victoria Hospital, Bahawalpur from 2015 to 2017 (*N* = 651)

Treatment outcomes	Patients n (%)	Total n (%)
**Favorable**		
Treatment completed	463 (71.1)	463 (71.1)
**Unfavorable**		
Treatment failure	2 (0.3)	
Died	10 (1.5)	177 (27.2)
Loss to follow-up	165 (25.4)	
**Not evaluated**	11 (1.7)	11 (1.7)

Further analysis of data showed that 20% of the patients with lymphatic TB lost to follow-up the treatment. While, 0.6% of the patients died during treatment. With regard to meningeal TB, 42.1% of the patients lost to follow-up and 2.6% of the patients died during the treatment. Further details on treatment outcomes in relation to patients' age, sex and site of infection could be seen in Supplementary File 1.

In simple logistic regression analysis, the factors associated with unfavorable treatment outcome included lymph node TB (OR 0.61, 95% CI 0.397, 0.922) and meningeal TB (OR 2.35, 95% CI 1.203, 4.605) (Table 5).

**Table 5:** Factors associated with unfavorable treatment outcome among extrapulmonary tuberculosis patients registered at Bahawal Victoria Hospital from 2015 to 2017: simple logistic regression analysis

Variables	Unfavorable outcome		OR	95% CI
	No n (%)	Yes n (%)		
**Female**				
No	240 (37.5)	88 (13.8)	1	–
Yes	223 (34.8)	89 (13.9)	1.08	.770–1.539
Age (years)*	–	–	1.0	.991–1.014
**Distance from home (Km)**				
< 5 km			1	
6 − 25 km	186 (29.1)	64 (10)	.84	.589–1.207
26 − 50 km	137 (21.4)	65 (10.2)	1.40	.959–1.989
> 50 km	69 (10.8)	32 (5)	1.26	.795–1.997
Body weight at start of treatment (Kg)*	–	–	1.0	.987–1.012
Body weight at end of treatment (Kg)*	–	–	.98	.946–1.024
**Pleural TB**				
No	316 (49.5)	111 (17.3)	1	
Yes	146 (22.8)	66 (10.3)	1.29	.899–1.855
**Lymphatic TB**				
No	329 (51.4)	142 (22.2)	1	
Yes	134 (20.9)	35 (5.5)	**.61**	.397–.922
**Abdominal TB**				
No	404 (63.1)	157 (24.5)	1	
Yes	59 (9.2)	20 (3.1)	.87	.509–1.496
**Bone/joint/spinal TB**				
No	448 (70)	172 (26.9)	1	
Yes	15 (2.3)	5 (0.8)	.87	.311–2.425
**Meningeal TB**				
No	443 (69.2)	160 (25)	1	
Yes	20 (3.1)	17 (2.7)	**2.35**	1.203–4.605
**Skin TB**				
No	460 (71.9)	174 (27.2)	1	
Yes	3 (0.5)	3 (0.5)	2.64	.529–13.223

*Entered as continuous variable

After adjusting the determinants of unfavorable treatment outcome among EPTB patients in univariate analysis, the factors which still remained significantly associated with unfavorable treatment outcome were lymph node TB (AOR 0.65, 95% CI 0.422, 0.989) and meningeal TB (AOR 2.1, 95% CI 1.065, 4.144) (Table 6).

**Table 6:** Factors associated with unfavorable treatment outcome among extrapulmonary tuberculosis patients registered at Bahawal Victoria Hospital, Bahawalpur from 2015 to 2017: multiple logistic regression analysis

Variable	Unfavorable outcome		AOR	95% CI
	No n (%)	Yes n (%)		
**Lymphatic TB**				
No	329 (51.4)	142 (22.2)	1	–
Yes	134 (20.9)	35 (5.5)	.65	.422–.989
**Meningeal TB**				
No	443 (69.2)	160 (25)	1	–
Yes	20 (3.1)	17 (2.7)	2.1	1.065–4.144

AOR = Adjusted odds ratio; Model summary (chi square = 10.208, degrees of freedom = 2, *p* = .006, pseudo R square = .023).

## Discussion

Our study findings showed the treatment success rate of 71.1% among the EPTB patients, which is far less than the target (i.e., ≥ 90%) set by the WHO. In the study cohort, more than 25% of the patients lost to follow-up, while 1.5% died during the treatment. Pleural and lymphatic TB were the most common forms of EPTB. Patients with meningeal TB had higher probability of unfavorable outcome, while the patients with lymphatic TB had relatively higher probability of achieving favorable treatment outcome.

A treatment success rate of ≥90% among TB patients is one of the main targets set in the End TB strategy [2]. In this study, treatment success rate among EPTB patients was suboptimal and this is in line with the findings of a previous Pakistani study which reported 60% treatment success rate among EPTB patients [14]. Other studies from high TB burden countries such as Nigeria (52.3%), India (78.1%) and China (76.7%) also reported suboptimal treatment success rates among EPTB patients [8, 9, 27]. The reasons for lower treatment success rate could be non-adherence to anti-TB drugs, potential side effects associated with anti-TB drugs, lack of patient knowledge about the consequence of loss to follow-up, and distance from treatment center [28]. Contrary to our findings, studies conducted in Ethiopia (89.2%) and Bhutan (90%) showed relatively better treatment success rates [12, 29]. This might be associated with better quality of healthcare facilities, political commitment, support from family, friends and healthcare workers, social support, better patient knowledge, and improved adherence to TB treatment [21].

In line with the findings of studies conducted in Cameroon (20%), South Africa (17.2%), Thailand (11%) and Pakistan (15.7%), our study found 25.4% loss to follow-up rate among EPTB patients [17, 30–32]. This loss to follow-up rate is substantially higher and needs urgent attention. Potential consequences of loss to follow-up could be treatment failure, relapse, transmission of disease to high risk patients, development of drug resistance, and mortality. A systematic review of studies from developing countries reported lack of patient knowledge about disease, getting bored of pills, longer duration of TB treatment, feeling better after few weeks of treatment, negative attitude of patients towards healthcare workers (HCW), poor patient-HCW relationship, fear of stigmatization, lack of money and social support, and distance from treatment center as main factors associated with loss to follow-up TB treatment [28]. Understanding and minimizing the impact of these factors is important to improve treatment completion rate. Improved patient counseling and awareness of disease, better healthcare facilities and social support to the patients can reduce loss to follow-up treatment.

In our study, the most common types of EPTB were pleural and lymph node TB. This finding is consistent with other published studies [33–35]. In multivarite binary logistic regression analysis, patients with meningeal TB had higher chances of unfavorable treatment outcome. This could be due the fact that treatment duration of meningeal TB is longer due to reduced penetration of anti-TB drugs (ethambutol and rifampicin) to cerebrospinal fluid [36, 37]. Consequently, they could have left the treatment earlier (i.e., loss to follow-up) either because they were bored of taking pills or were unaware of the importance of treatment completion [28]. Secondly, due to earlier reasons and complex nature of the disease, the mortality rate may be higher in patients presenting with meningeal TB [38]. Our data support this notion as 42% of the patients with meningeal TB lost to follow-up and 2.6% of the patients died during the treatment. Earlier studies conducted in the United States (US) and Iran also reported meningeal TB to be a risk factor associated with unsuccessful outcome among EPTB [39, 40]. Our findings also showed that probability of unfavorable treatment outcome was less in patients with lymph node TB. This could be attributed to relatively lower loss to follow-up (20%) and death rates (0.6%) in this sub group of patients. A study from the US highlighted better success rate among lymph node TB patients due to lower mortality rate, and this is also evident from our findings [38].

The strength of this study was that we included all patients registered at the study site during the study period and reported outcomes of TB treatment in line with the standard WHO criteria. Similarly, design, methodology and reporting of results followed STROBE guidelines [41]. Therefore, the risk of methodological biasness was minimal. Along with the strengths, there are a few limitations with are innate to any operational research study. First, the findings of this study could not be generalized for whole Pakistan. This is because BVH is one of the main referral hospital in the Southern Punjab and serve complex TB cases from urban and rural areas. The patients from rural areas may have higher probability of loss to follow-up, and this is also evident from our findings. Second, due to incomplete medical records of patients, it was not possible to capture some of the important variables which could have influenced the treatment outcome.

This study has implications for policy and practice. The findings of the study demonstrated that treatment success (favorable treatment outcome) rate among EPTB patients was less than the target set in the End Treatment Strategy which warrants urgent attention. In this study, a large proportion of EPTB patients lost to follow-up. This should be a concern for the policy makers and it necessitates the development and implementation of interventions to minimize treatment interruptions. Moreover, future studies should be conducted to underline the reasons of loss to follow-up. Our study also provided clinicians an opportunity to identify patients who were at higher risk of unfavorable treatment outcome.

## Conclusion

Higher proportion of patients had pleural TB followed by lymph node TB. The treatment success rate (favorable outcome) among EPTB patients was 71.1% which is less than the target (i.e., ≥ 90%) set by the WHO in the End TB Strategy. Among the unfavorable treatment outcomes, highest proportion of patients lost to follow-up the treatment. Patients with meningeal TB had higher probability of unfavorable outcomes, while patients with lymph node TB were more likely to achieve favorable treatment outcome.

### Ethical approval and consent to participate

The ethical clearance of the study was obtained from the Pharmacy Research Ethics Committee (PREC) at the Islamia University of Bahawalpur.

## Supplementary information

**Additional file 1.**

## Data Availability

The raw data related to this study may be provided upon receiving reasonable request. Please contact Muhammad Atif at pharmacist_atif@yahoo.com.
