# Supplementary material for: Treatment outcomes of extrapulmonary tuberculosis in Bahawalpur, Pakistan; a record review
Source: J Pharm Policy Pract. 2020 Jul 24;13:35. doi: 10.1186/s40545-020-00227-1 (PMC7382058; doi:10.1186/s40545-020-00227-1)
Supplement: Supplementary file 1 — Additional file 1. [file 40545_2020_227_MOESM1_ESM.docx]

**Supplementary File 1**

Treatment outcome in relation to sex, age and site of infection among extrapulmonary tuberculosis patients registered at Bahawal Victoria Hospital, Bahawalpur from 2015–2017

| **Characteristics** | **Patients**  **n** | **Treatment completed n (%)** | **Treatment failure n (%)** | **Died n (%)** | **Loss to follow-up n (%)** | **Not evaluated n (%)** |
| --- | --- | --- | --- | --- | --- | --- |
| **Sex** |  |  |  |  |  |  |
| Male | 334 | 240 (71.9) | 1 (0.3) | 5 (1.5) | 82 (24.6) | 6 (1.8) |
| Female | 317 | 223 (70.3) | 1 (0.3) | 5 (1.6) | 83 (26.2) | 5 (1.6) |
| **Age group (years)** |  |  |  |  |  |  |
| 15−24 | 232 | 168 (72.4) | 0 (0) | 3 (1.3) | 55 (23.7) | 6 (2.6) |
| 25−34 | 172 | 115 (67.3) | 0 (0) | 3 (1.8) | 48 (28.1) | 5 (2.9) |
| 35−44 | 92 | 66 (71.7) | 2 (2.2) | 0 (0) | 24 (26.1) | 0 (0) |
| 45−54 | 75 | 61 (81.3) | 0 (0) | 0 (0) | 14 (18.7) | 0 (0) |
| 55−64 | 45 | 30 (66.7) | 0 (0) | 2 (4.4) | 13 (28.9) | 0 (0) |
| ≥ 65 | 36 | 23 (63.9) | 0 (0) | 2 (4.4) | 11 (30.6) | 0 (0) |
| **Site of infection** |  |  |  |  |  |  |
| Pleural | 217 | 146 (67.3) | 1 (0.5) | 7 (3.2) | 58 (26.7) | 5 (2.8) |
| Lymphatic | 170 | 134 (78.8) | 0 (0) | 1 (0.6) | 34 (20) | 1 (0.6) |
| Abdominal/ascites | 80 | 59 (73.8) | 0 (0) | 0 (0) | 20 (25) | 1 (1.3) |
| Bone/joint/spinal | 23 | 15 (65.2) | 1 (4.3) | 0 (0) | 4 (17.4) | 3 (13) |
| Meningeal | 38 | 20 (52.6) | 0 (0) | 1 (2.6) | 16 (42.1) | 1 (2.6) |
| Skin | 6 | 3 (50) | 0 (0) | 0 (0) | 3 (50) | 0 (0) |
| Other ^a^/not recorded | 117 | 86 (73.5) | 0 (0) | 1 (0.9) | 30 (25.6) | 0 (0) |

^a^ Testicular, Genitourinary, other organ involvement
